# A controlled pilot trial of a nurse-led intervention (Mini-AFTERc) to manage fear of cancer recurrence in patients affected by breast cancer

**DOI:** 10.1186/s40814-020-00610-4

**Published:** 2020-05-07

**Authors:** Calum T. McHale, Susanne Cruickshank, Claire Torrens, Jo Armes, Deborah Fenlon, Elspeth Banks, Tom Kelsey, Gerald M. Humphris

**Affiliations:** 1 Division of Population and Behavioural Sciences, School of Medicine, University of St Andrews, St Andrews, KY16 9TF UK; 2 Faculty of Health Sciences and Sport, University of Stirling, Stirling, UK; 3 School of Health Sciences, University of Surrey, Guildford, Surrey UK; 4 College of Human and Health Sciences, Swansea University, Swansea, UK; 5 National Cancer Research Institute, London, UK; 6 School of Computer Science, University of St Andrews, St Andrews, UK

**Keywords:** Breast cancer, Psychological, Fear of cancer recurrence, Feasibility, Brief intervention

## Abstract

**Background:**

Fear of cancer recurrence (FCR) is common in people affected by breast cancer. FCR is associated with increased health service and medication use, anxiety, depression and reduced quality of life. Existing interventions for FCR are time and resource intensive, making implementation in a National Health Service (NHS) setting challenging. To effectively manage FCR in current clinical practice, less intensive FCR interventions are required. Mini-AFTERc is a structured 30-min counselling intervention delivered over the telephone and is designed to normalise moderate FCR levels by targeting unhelpful behaviours and misconceptions about cancer recurrence.

This multi-centre non-randomised controlled pilot trial will investigate the feasibility of delivering the Mini-AFTERc intervention, its acceptability and usefulness, in relation to specialist breast cancer nurses (SBCNs) and patients. This protocol describes the rationale, methods and analysis plan for this pilot trial of the Mini-AFTERc intervention in everyday practice.

**Methods:**

This study will run in four breast cancer centres in NHS Scotland, two intervention and two control centres. SBCNs at intervention centres will be trained to deliver the Mini-AFTERc intervention. Female patients who have completed primary breast cancer treatment in the previous 6 months will be screened for moderate FCR (FCR4 score: 10‑14). Participants at intervention centres will receive the Mini-AFTERc intervention within 2 weeks of recruitment. SBCNs will audio record the intervention telephone discussions with participants. Fidelity of intervention implementation will be assessed from audio recordings. All participants will complete three separate follow-up questionnaires assessing changes in FCR, anxiety, depression and quality of life over 3 months. Normalisation process theory (NPT) will form the framework for semi-structured interviews with 20% of patients and all SBCNs. Interviews will explore participants’ experience of the study, acceptability and usefulness of the intervention and factors influencing implementation within clinical practice. The ADePT process will be adopted to systematically problem solve and refine the trial design.

**Discussion:**

Findings will provide evidence for the potential effectiveness, fidelity, acceptability and practicality of the Mini-AFTERc intervention, and will inform the design and development of a large randomised controlled trial (RCT).

**Trial registration:**

ClinicalTrials.gov: NCT0376382. Registered 4th December 2018, https://clinicaltrials.gov/ct2/show/NCT03763825

## Background

Fear of cancer recurrence (FCR) is prevalent amongst people affected by breast cancer, with 60% or higher claiming they want to speak with their cancer care team to discuss their fears [1]. Fear of cancer recurrence is defined as “Fear, worry, or concern relating to the possibility that cancer will come back or progress” [2]. FCR is an understandable reaction to surviving a serious illness; however, when it becomes severe it can produce unwanted effects including loss of concentration, lowered quality of life, propensity to depression as well as increased health service utilisation, medication and over frequent self-examination [3–6]. FCR results from the activation of certain cognitions, beliefs and emotions that an individual acquires following cancer diagnosis and treatment [7]. Therefore, psychological interventions are needed to assist patients to better cope with FCR [6].

Intensive psychological interventions for FCR are available, including ConquerFear [8], SWORD [9] and AFTER [10]. However, such interventions are designed for those with ‘severe’ FCR and are highly resource and time intensive. In the UK, a national lack of funding and highly trained mental health professionals (psychologists, psychiatrists, etc.) across the National Health Service (NHS) [11] would make implementing such intensive interventions difficult. Nevertheless, some form of intervention is crucial as once FCR becomes severe, it is particularly challenging to reduce [6]. Interventions may also reduce costs and pressure on specialist services because patients with high FCR use more specialist services [12, 13].

A recent randomised control trial of an online-only intervention (CAREST-trial) to support individuals to manage elevated FCR found that the intervention produced no significant reduction in FCR [14]. This finding demonstrates that online self-management approaches may not effectively manage FCR and highlights the potential need for interpersonal interaction to improve FCR. A review of FCR interventions delivered by non-mental health professionals concluded that professionals other than psychologists can deliver psychological and counselling interventions to reduce FCR in patients affected by cancer [15]. Specialist cancer nurses are potentially ideal candidates to deliver FCR interventions as they provide psychological support to patients from the point of cancer diagnosis onwards [15]. Given this evidence, it is important that further research is undertaken to develop and test less intensive FCR interventions that can be delivered by non-mental health professionals and are suitable to implement into current clinical practice.

The Mini-AFTERc intervention is a cognitive behavioural intervention based on the self-regulation model (SRM) of illness cognitions [16]. It is a less intensive version of the AFTER intervention [10] and is designed to be delivered by trained specialist cancer nurses in approximately 30 min [17]. Mini-AFTERc aims to reduce FCR in people affected by cancer by targeting inappropriate or unhelpful behaviours (e.g. twice daily self-examination), and misconceptions about cancer recurrence. A common example of misconception is that patients will interpret new symptoms or sensation, such as acute pain, numbness or tingling, as cancer recurrence. Before the cancer diagnosis, patients may have interpreted these new experiences as harmless, but post-diagnosis patients may assume that they have sinister connotations. The intervention does not intend to dispel fears entirely or to reduce unduly attention to symptom change and appropriate self-examination behaviours.

A feasibility study of the Mini-AFTERc intervention was conducted within one breast service in NHS Scotland [17]. A sample of 16 patients who had completed breast cancer treatment reported a significant decrease in FCR at 1 week after the intervention was delivered. In this setting, specialist breast cancer nurses (SBCNs) who were trained in and delivered Mini-AFTERc found the intervention manageable and easy to follow. SBCNs also believed the intervention may be useful for reducing the demand on their time and resources by addressing FCR before it becomes severe [17]. Additionally, a mixed-methods study with 90 UK-based SBCNs aimed to determine the acceptability of implementing the Mini-AFTERc intervention into current practice [18]. SBCNs were asked how FCR was currently identified, assessed and supported, and their willingness to implement the Mini-AFTERc, via an online survey and semi-structured interviews. FCR was reported to be an important issue by SBCNs, but their reported practices to identify, assess and support FCR were highly variable. SBCNs viewed the Mini-AFTERc intervention favourably and believed it would enhance their skills in the managing FCR [18]. Following the favourable outcomes of these studies, additional research is required to assess the feasibility and acceptability of the Mini-AFTERc intervention amongst a larger sample of patients.

## Methods

### Aim and objectives

The aim of this pilot trial is to investigate the acceptability and feasibility of delivering the Mini-AFTERc intervention in every day clinical practice. The objectives are as follows:

1. To develop a procedure for training SBCNs in the delivery of the Mini-AFTERc intervention.
2. To assess the acceptability of the Mini-AFTERc intervention for SBCNs and patients and the feasibility of introducing the intervention into current NHS service provision.
3. To apply a formal decision-making framework to identify potential difficulties in the implementation of the pilot trial and appraise solutions prior to the development of a full-randomised control trial.

### The Mini-AFTERc intervention

The Mini-AFTERc intervention is a manualised structured discussion plan comprising of five topics which are discussed during a 30-min telephone discussion. The Mini-AFTERc intervention was designed to target specifically the antecedents within the cognitive formulation of FCR developed by Lee-Jones et al. [7].

**Fig. 1:**
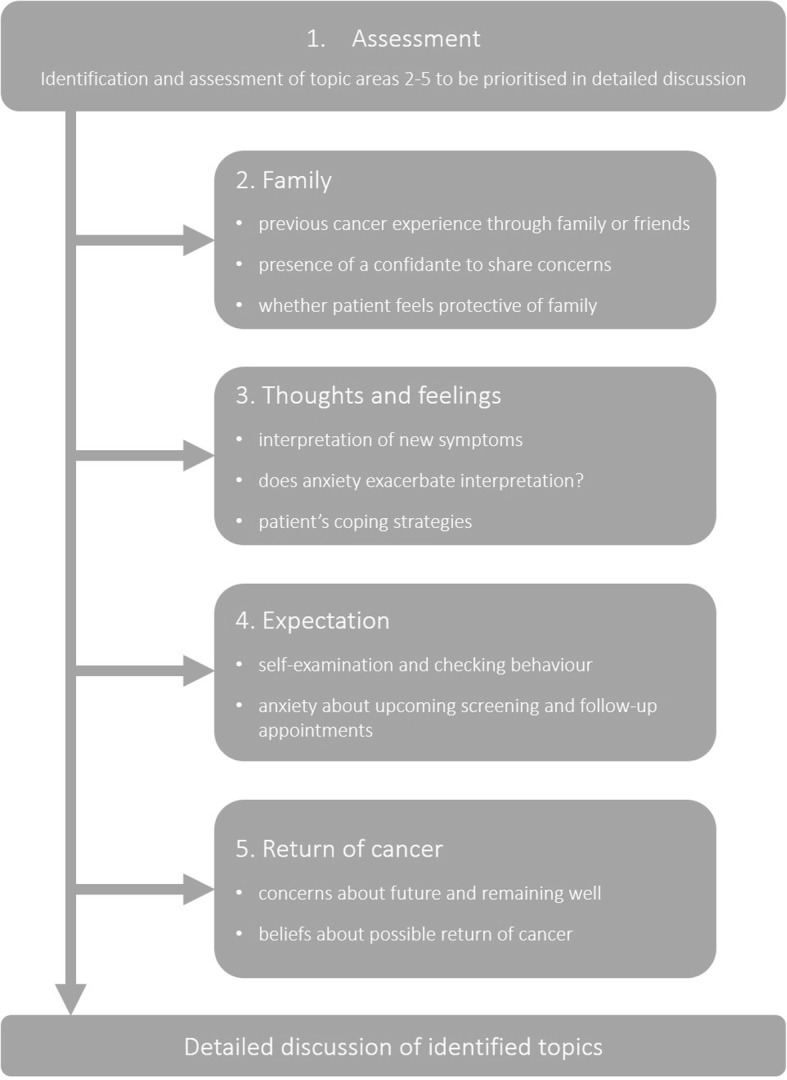
Topics covered during the Mini-AFTERc assessment phase

The first stage of the Mini-AFTERc intervention is assessment. The assessment stage aims to establish an understanding of the participant’s experiences and symptoms of FCR and to tailor the subsequent intervention discussion to the participant’s needs. Assessment processes are common to all cognitive behavioural-based interventions [19]. The Mini-AFTERc assessment is structured (Fig. 1) to ensure that all aspects of the four primary discussion topics (family, thoughts and feelings, expectations, return of cancer) are raised with the participant. This allows the individual delivering the intervention to prioritise and target one or two of the discussion topics for further detailed discussion.

Family is the first detailed discussion topic of the Mini-AFTERc intervention. Social constraints have been shown to increase FCR [20] and present a possible pathway to FCR development, therefore this topic focuses on communication processes with the participant’s family and friends. The quality of their participant’s relationship with family and friends is assessed and whether the participant has an individual who they can have open discussions with about their thoughts and feelings (a confidante). The individual delivering the intervention may encourage the participant to have more open discussion with family and friends about how they are feeling and/or to explore other means of social support (e.g. cancer support groups).

The second detailed discussion topic is thoughts and feelings. This topic concentrates on the identification of symptoms experienced during treatment and those after treatment completion. This phenomenon has been identified previously in people affected by breast cancer [21]. Participants who associate symptoms (i.e. ‘triggers’) as indicators of cancer returning are invited to describe these symptoms in greater detail. The individual delivering the intervention may normalise the participants’ experiences (i.e. “many people experience these symptoms after treatment”); an approach which has been used effectively in the field of psychosis [22]. They may also provide additional information to persuade the participant that such symptoms are not necessarily indicative of cancer returning [23].

The third detailed discussion topic is expectations. This topic invites participants to relate their concerns about follow-up visits, scans and self-awareness/checking. Previous evidence indicates that expectations about the outcome of upcoming out-patient visits [24] and/or self-examination behaviours [21] may heighten FCR. Some individuals may engage in these behaviours as an FCR management strategy, conforming to a cognitive behavioural model. The individual delivering the intervention may assist participants to understand their reactions to their behaviour [19] and recommend graded behaviour change.

The fourth discussion topic is return of cancer and focuses on the participants thought about the future. The individual delivering the intervention may normalise the participants’ concerns about the future. Through a process of brief graded exposure [25] to the possibility of the cancer returning, they may encourage the participant to express their anxieties openly.

The Mini-AFTERc discussion concludes with a short summary of issues covered and suggested recommendations or action plans discussed during the telephone conversation. Participants will also be given the opportunity to ask any questions they may have. Thirty minutes is considered the optimal time required to fully address two of the discussion topics. Nurses will be advised to complete the discussion within 30 min however this is not categorical. Nurses will be encouraged to use their discretion to extend the discussion beyond 30 min. The duration of discussions will be recorded as part of this study. If the nurses have any outstanding concerns about a patient after their discussion that they think require more extensive discussion then they are advised to refer the patient for additional psychological support through the usual pathway at their cancer centre.

Patient FCR will be monitored after the intervention has been delivered as part of this study using a self-report questionnaire. Any patients who display increasing FCR will be offered additional support at the end of the study. This support may include one-to-one counselling at the breast cancer centre or the offer of a referral to specialist psychological services. All referrals and offers of additional support will be recorded as part of this pilot trial.

### Trial design

This study is a non-randomised controlled pilot trial conducted within four breast cancer centres in NHS Scotland. The Mini-AFTERc intervention will be delivered at two of the sites in addition to standard care and compared to standard care alone delivered at the two control sites. The selection of cancer centres was dependant on the proximity of the research institutions and local knowledge of service provision. Guidance in relation to breast cancer follow-up after primary diagnosis is inconsistent [18]. Consequently, standard care (i.e. follow-up and support for patients once they have completed primary cancer treatment) can vary between cancer centres, in terms of timing and types of support offered. SBCNs at the intervention sites will be recruited and trained to deliver the Mini-AFTERc intervention using a pre-determined training programme, which will be further developed and refined with feedback from participating SBCNs during this trial.

**Fig. 2:**
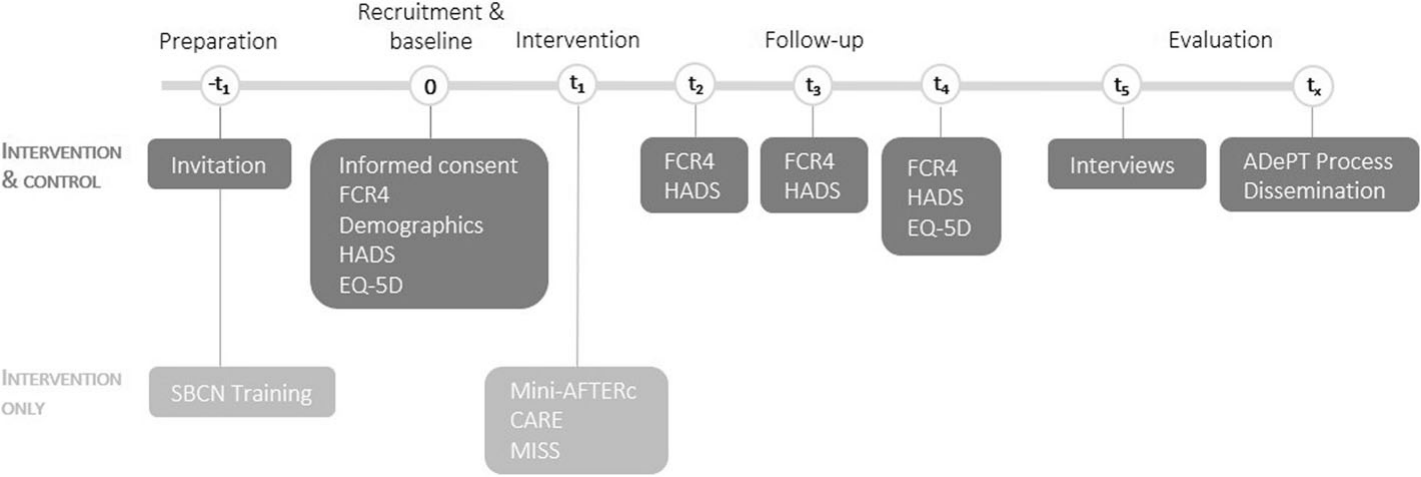
Mini-AFTERc pilot trial timeline, following SPIRIT [26]

Participants will be recruited from hospital clinics and over the telephone. Participants from intervention centres will receive an intervention telephone call within 2 weeks of recruitment. All participants (intervention and control) will be asked to report their FCR, mood and quality of life over a three-month follow-up period (Fig. 2). A bespoke smartphone application, already designed to collection questionnaire responses form patients, will also be tested as part of this pilot trial.

Patient involvement is a core principle in the design and implementation of this pilot trial. Elspeth Banks (EB) is a representative of the National Cancer Research Institute (NCRI) and the Independent Cancer Patients’ Voice (ICPV). EB was regularly consulted during the design of this pilot trial and on the acceptability of this research from a patient’s perspective. EB will continue to be a key partner throughout the implementation of this trial and the analysis of the findings.

This pilot trial will be conducted in three consecutive phases: preparatory phase, intervention delivery and data collection phase, and evaluation phase (Table 1).

**Table 1:** Phases of the Mini-AFTERc pilot trial

Phase	Overview of procedures
Phase 1: Preparatory	• Development of SBCN intervention training programme • Delivery of training programme via a workshop for consenting SBCN in intervention centres • Development and testing of Mini-AFTERc smartphone application
Phase 2: Intervention delivery and data collection	• Screening and recruitment of patients at all centres • Intervention delivery at intervention centres • Collection of patient demographic and outcome data via smartphone app at baseline and 3 follow-up time points • Semi-structured interviews with SBCNs and sample of patient participants
Phase 3: Evaluation	• Statistical analysis of outcome data • Fidelity analysis of SBCNs adherence to intervention • Use of NPT in analysis of patient and nurse semi-structured interviews • Trial evaluation following the ADePT process

### Phase 1: Preparatory

The purpose of phase 1 is to develop the Mini-AFTERc intervention training programme for SBCN and to deliver the training at cancer centres that will be delivering the intervention. Additionally, a bespoke Mini-AFTERc smartphone application for the collection of research data will be developed and piloted during phase 1.

#### Mini-AFTERc intervention training

A detailed Mini-AFTERc training manual was developed for the previous Mini-AFTERc feasibility study [17]. This manual provides SBCNs with a detailed overview of the theory and structure of the Mini-AFTERc intervention, as well as providing examples of questions and prompts to aid SBCNs to facilitate intervention discussions with patients. The planned structure of the training sessions is as follows:

*Session 1*. Present an overview of the Mini-AFTERc intervention, fear of cancer recurrence and the purpose of the phone call. Trainees will be provided with a detailed explanation of the training manual, including the structure of Mini-AFTERc intervention and transcript examples of how to deliver the different stages of the intervention from previously recorded Mini-AFTERc telephone discussions (from the previous feasibility study [17]).

*Session 2*. An interactive workshop using roleplay scenarios representing each section of the Mini-AFTERc intervention. For each scenario, the initial stage is scripted and then SBCNs continue the conversation unscripted according to the Mini-AFTERc principles. SBCNs can “pause” and “restart” the discussion at any point to confer, clarify or reflect on their conversations and gain support and guidance from the trainers. This session also provides SBCNs with training in the use of the recording equipment and data collection requirements prior to beginning a Mini-AFTERc discussion.

*Session 3*. This session aims to simulate the Mini-AFTERc telephone discussion by using a patient expert and use of the recording equipment. Case studies have been written for the simulated patient. These scenarios were based on the clinical experience of GH. SBCNs deliver the Mini-AFTERc intervention as they would in their clinical practice setting; in a private room, over the telephone and using the audio recording devices. The SBCNs will receive a debrief from the lead researcher. Following a review of these recordings, a future meeting will be organised with each nurse participant to provide feedback.

All training sessions will be delivered by the research team based in Scotland. Following the formal training sessions, SBCNs will be offered continued support via email and telephone from the research team for the duration of the trial. Nurses will have frequent interaction with the research team during the trial and will be encouraged to feedback any challenges or difficulties they encounter during the trial. The research team will work with the nurses to find a suitable solution.

#### Smartphone application development

**Fig. 3:**
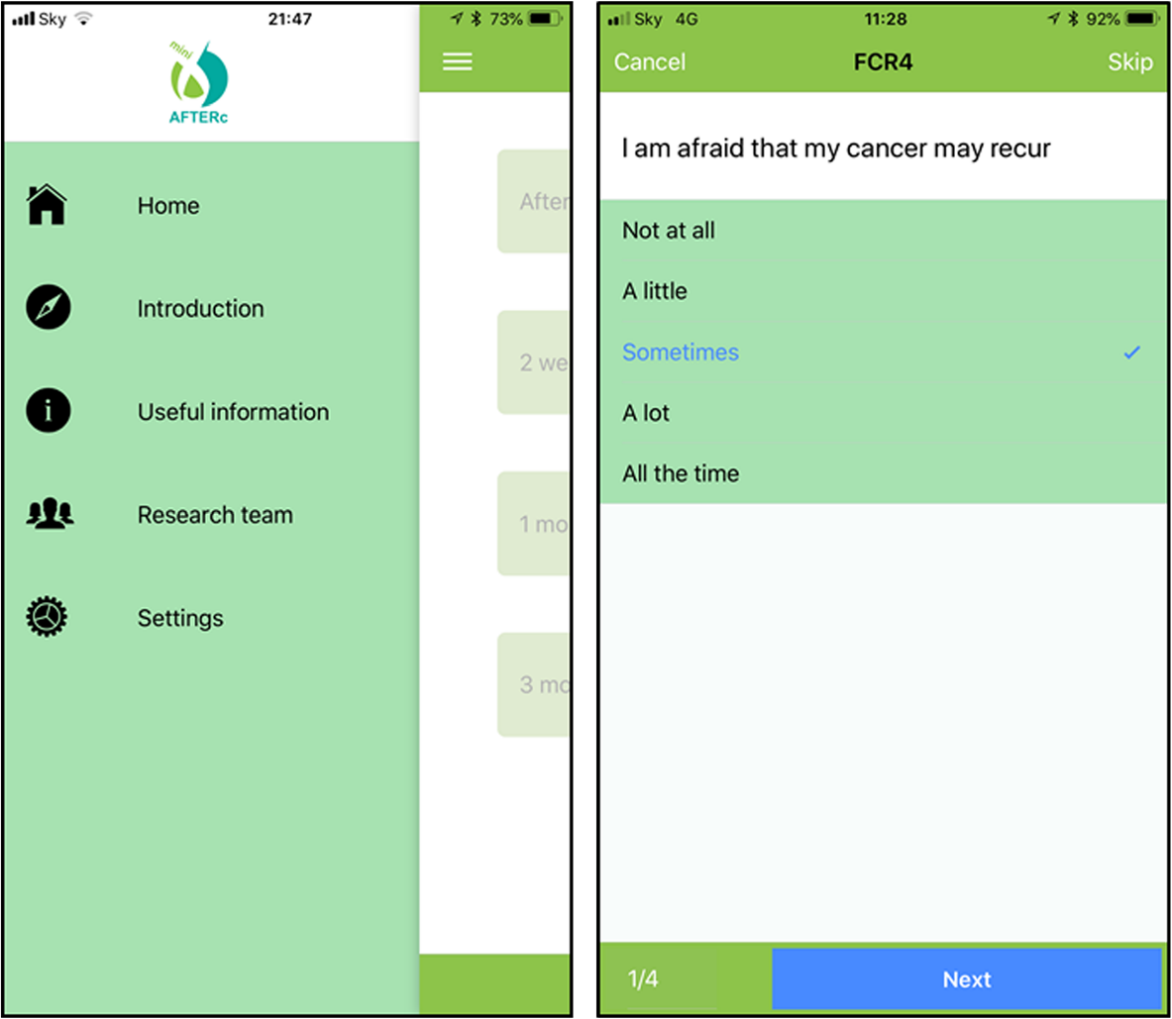
Screen captures of prototype Mini-AFTERc smartphone application

A bespoke smartphone application (app) will be designed to support data collection during this pilot trial. The app will be collaboratively developed by research team members in the Schools of Medicine and Computer Science at the University of St Andrews. The app provides a secure site for participants to electronically submit questionnaire responses throughout the trial. The app will send notifications to participants to prompt them to complete questionnaires at the pre-defined time points. A prototype of the app has been developed (Fig. 3). Early prototypes have been reviewed by the patient expert on the research team (EB) and informed its development. Throughout phase 1, the design and functionality will be continuously reviewed by all members of the research team.

### Phase 2: Intervention delivery and data collection

#### Eligibility and recruitment

Mini-AFTERc is designed for patients with *moderate* FCR, therefore consented patients will complete the fear of cancer recurrence 4-item (FCR4) measure [27] to determine their eligibility (score range: 10‑14). The FCR4 has been previously shown to be reliable (*α* 0.93, 95% CI 0.91‑0.94) [27]. Any patient who score > 14 (i.e. high) on the FCR4 will be offered a referral onto psychological support services, in line with standard practice at each cancer centre. Additional patient eligibility criteria for screening are broad to ensure external validity of the sample. Patients will be eligible for screening if (1) they have completed their primary cancer treatment for breast cancer, (2) are cancer-free, (3) are female, (4) are aged 18 or over and (5) their responsible clinician agrees to their participation. Patients will be ineligible for screening if they have not completed their cancer treatment, are not cancer-free, are male and/or are receiving treatment for a diagnosed psychotic disorder that is known to the cancer service.

Potential participants will be identified in the cancer centres via patient appointment systems. A one-page study information letter will be sent to potential participants prior to their follow-up appointment or end of treatment review. There are two potential recruitment and consent processes:

*Process 1*. Participants attending in person will be invited to speak directly with a researcher about the study in the clinic. This meeting will provide study information and consent will be taken to participate in the trial.

*Process 2*. Participants not attending in person will have the study introduced by their cancer care team and be asked to consent to their contact details being shared with the research team. The researchers will contact potential participants to discuss the trial, provide additional information and consent. All study information and consent forms will be mailed. Consent forms will be required to be returned before participants can continue in the study.

#### Sample size

The trial aims to recruit 130 breast cancer patients, approximately 33 patients at each breast cancer centre. Researchers will screen patients until 65 patients have been recruited to the intervention group and 65 patients to the control group. Screening will continue until the target sample size has been reached. The anticipated recruitment period will take place over 12 months. An effect size of 0.5 at 0.85 power would require a sample of 130 patients to robustly demonstrate any effect of the intervention on patients’ fear of cancer recurrence, assuming a standard deviation of 7 between pre and post FCR measures, derived from a previous feasibility study [17], and an attrition rate of 30%.

#### Screening and baseline assessment

Screening will be conducted with the fear of cancer recurrence 4-item (FCR4) measure [27]. Participant demographical information, including age (in years), cancer treatments received, education, employment and living situation, will also be collected during screening but eligibility will not depend on this information. A score of ≥ 10 and < 15 in the FCR4 is defined as ‘moderate’ FCR. Patients will be excluded from the trial if they score ‘low’ (< 10) or ‘high’ (≥ 15) on the FCR4. It is anticipated that 3 in 10 patients will obtain an FCR4 scoring in the moderate range (60th percentile) therefore approximately 440 participants will be required to be screened to obtain the required sample of 130. If eligible after screening, participants will be asked to complete the Hospital Anxiety and Depression Scale (HADS) [28] and the Euro-Qol 5-dimension health-related quality of life measure (EQ-5D-5 L) [29] at baseline. All initial screening and baseline data collection will be completed by the researcher and the participant either face to face or over the telephone.

#### Intervention delivery

Patients will receive the Mini-AFTERc intervention telephone call from one of the trained breast care nurses within 2 weeks of screening. Patients will be given a date and time for the telephone call from the researcher on the day of screening. Telephone calls will last approximately 30 min and will be audio recorded by the nurse. Nurses may have consulted patients during their treatment and have access to their records during the intervention discussion.

#### Follow-up assessment

Participants in the intervention group will complete the consultation and relational empathy (CARE) [30] and the medical interview satisfaction scale (MISS) [31] within 1 or 2 weeks following their telephone discussion to measure acceptability. Additionally, the intervention group will complete the HADS and EQ-5D measures at 2 weeks, 1 month and 3 months following the intervention telephone discussion. Participants in the control group will complete the same measures at 3 weeks, 5 weeks and 13 weeks following baseline. The minor time point differences are to ensure data are collected at a similar time point in the study for intervention and control groups, allowing time for the intervention to be delivered. See supplementary file 1 for a comprehensive overview of all patient outcomes assessed in with research questionnaires.

All follow-up measures will be accessed by patients using the smartphone app. The app will be installed on the participants’ mobile phone after recruitment. To accommodate participants who cannot attend in person or do not have a compatible smartphone, a web browser version of the app has been developed or paper versions of the questionnaires can be made available. Alternative arrangements have been put in place to distribute paper copies at the different time points, due to technical difficulties with the smartphone application or participant preference.

#### Semi-structured interviews

Interviews with patients will cover their experiences during the recruitment process and during data collection, including timing and methods of collection (mobile phone app or paper-based). Patients in the intervention group will be asked about the usefulness and impact of the Mini-AFTERc intervention, including the method of delivery (telephone call), and ways to improve the intervention. Interviews with SBCNs will explore their views on the usefulness and impact of the intervention and areas where more help was required.

A 20% sample of patients (approximately 26) will be interviewed. A sampling matrix (see supplementary file 2) will ensure a representative sample of patients is recruited based on age, research site, trial group (control/intervention), and retention post-screening (attended/ dropped-out). All breast cancer nurses who delivered the intervention will also be invited for an interview.

### Phase 3: Evaluation

#### Quantitative analysis

Data collected from self-report questionnaires will be quantitatively analysed using the STATA software [32]. A detailed CONSORT diagram will be completed with fully annotated reasons for withdrawal from the study and follow-up rates where possible. This will also include numbers referred onto further specialist services during the study. Descriptive statistics of questionnaire data will be reported and inferential statistics, including mixed regression modelling, will investigate outcome variable associations. Growth curve analysis (linear and polynomial parameters), will be performed using MPlus [33] to calculate the fear of cancer recurrence trajectory and intercept link intervention and control groups. Interclass correlation coefficient (ICC) will also be determined to estimate the potential power required for future trials, based on the number of clusters and sample size per cluster. The Monte Carlo modelling approach within MPlus, advanced by Bolger and Laurenceau [34], will be utilised to determine the sample size required to estimate for a moderate effect of the difference in trajectory (i.e. slope coefficient) between intervention and control groups with covariates (age, treatment experience, disease severity). Alpha set to 0.05 (2-sided). Analysis will not be blinded and will be conducted by the local research teams (St Andrews and Stirling Universities) who will seek advice from a divisional statistician (St Andrews).

#### Fidelity of intervention

A fidelity measure has been designed to assess the SBCNs’ adherence to the Mini-AFTERc intervention manual [35]. The fidelity measure will be applied to transcripts of the Mini-AFTERc intervention the audio recordings collected during the trial. The fidelity measure assesses adherence to key components (e.g. duration of discussion) of the intervention and scoring is informed by the principles of therapeutic alliance [36].

#### Qualitative analysis of interviews

The primary aim of the interviews is to assess the acceptability of the intervention to participants and the feasibility of incorporating the intervention into current NHS service provision. All interviews will be recorded on digital recorders and transcribed verbatim.

The interview schedule and analysis will be informed by normalisation process theory and its four main tenets: coherence, cognitive participation, collective action and reflexive monitoring [37]. It provides a constructive approach to explain and evaluate the underlying mechanisms of the implementation process and the components used to provide information for the optimisation of trial parameters [37]. The research team has experience of using NPT in the area of FCR among nurses [18]. However, further adaptation will be undertaken to align the component parts for the participant interviews.

#### Pilot trial evaluation

A process of decision-making after pilot and feasibility trials (ADePT) [38] will be used to evaluate the evidence from this pilot trial. The 3 key steps of ADePT facilitate the systematic appraisal of pilot and feasibility trials to identify all potential problems and solutions to these problems prior to the expansion of the trial. Findings from the quantitative data and analysis of interview data, from the perspective of patients and SBCNs, will support the decision-making process.

## Discussion

The Mini-AFTERc intervention has been designed to provide current cancer care professionals with a structured framework for discussing FCR with patients and assisting patients to address and reduce elevated levels of FCR at the end of primary cancer treatment. To our knowledge, Mini-AFTERc is the first brief FCR intervention, designed to assist individuals to manage moderate levels of FCR before they progress to more severe level and require a more intensive face-to-face psychological intervention. Preliminary feasibility work is promising, suggesting that Mini-AFTERc can feasibly be integrated into existing end-of-treatment cancer care practice, is acceptable to current cancer care staff and can effectively reduce FCR in patients affected by breast cancer [17, 18]. The proposed pilot trial will allow us to expand this feasibility work and determine the optimal approach to assess Mini-AFTERc in a controlled trial format.

SBCNs working in the UK recognise FCR as a primary concern of patient affected by cancer and many express a desire to receive training and develop the skills to better support patients with FCR [18]. The Mini-AFTERc intervention training programme has been specifically designed for SBCNs, to complement and develop the supportive skills that they already possess and have developed through routine practice. This pilot trial aims to further refine the Mini-AFTERc intervention training programme by allowing participating SBCNs to determine the focus of training sessions and provide feedback to the research team. Through this approach, we intend to develop a comprehensive but flexible training programme that can be appropriately and usefully delivered to SBCNs of different skill levels and across different centres.

Our feasibility work has determined that SBCNs may find the Mini-AFTERc intervention feasible and acceptable; however, we have yet to more broadly determine how patients may react to Mini-AFTERc. Involving patients at an early stage in research design is known to enhance the quality and appropriateness of research [39]. In keeping with our commitment to patient involvement in the development of this protocol, we intend to conduct comprehensive interviews with a representative sample of patients who participate in this trial. These interviews will explore patient thoughts about the Mini-AFTERc intervention, in terms of acceptability and usefulness, as well as their perceptions of participating in the trial. By considering and combining patient and nurse interviews with a structured decision-making framework (ADePT), we intend to refine the Mini-AFTERc intervention in addition to the trial structure and processes.

With this pilot work, we intend to collect the necessary data to design a large-scale randomised controlled trial of the Mini-AFTERc intervention. We believe this information will be beneficial for and of interest to patients affected by cancer as well as cancer services. We expect that this pilot trial will demonstrate that Mini-AFTERc is a feasible intervention that can be widely integrated into current cancer care practice and provide much needed emotional support to patients who have reached the end of cancer treatment.

## Supplementary information

**Additional file 1.** Patient outcome measures used in the Mini-AFTERc pilot trial

**Additional file 2.** Sampling matrix for semi-structured interviews

## Data Availability

Not applicable.

## Abbreviations

ADePT: A process of Decision-making after Pilot and feasibility Trials
AFTER: Adjustment to the Fear, Threat or Expectation of Recurrence intervention
CARE: Consultation and Relational Empathy measure
CAREST-Trial: Cancer Recurrence Self-help Training Trial
CONSORT: Consolidated Standards of Reporting Trials
EQ-5D-5 L: EuroQol 5 Dimension Measure of Health-related Quality of Life
FCR: Fear of cancer recurrence
FCR4: Fear of cancer recurrence 4-item measure
HADS: Hospital anxiety and depression scale
ICC: Inter-class correlations
ICPV: Independent cancer patients’ voice
Mini-AFTERc: Adjustment to the Fear, Threat or Expectation of cancer Recurrence - brief (mini) intervention
MISS: Medical interview satisfaction scale
NCRI: National Cancer Research Institute
NHS: National Health Service
NPT: Normalisation process theory
RCT: Randomised controlled trial
SBCN: Specialist breast cancer nurses
SRM: Self-regulation model
SWORD: Survivors’ worries of recurrent disease
