# Supplementary material for: A controlled pilot trial of a nurse-led intervention (Mini-AFTERc) to manage fear of cancer recurrence in patients affected by breast cancer
Source: Pilot Feasibility Stud. 2020 May 7;6:60. doi: 10.1186/s40814-020-00610-4 (PMC7204012; doi:10.1186/s40814-020-00610-4)
Supplement: Supplementary file 1 — Additional file 1. Patient outcome measures used in the Mini-AFTERc pilot trial [file 40814_2020_610_MOESM1_ESM.docx]

Supplementary file 1: Patient outcome measures used in the Mini-AFTERc pilot trial

| **Measure** | **Description** | **Properties and validity** |
| --- | --- | --- |
| FCR4 (1) | Fear of cancer recurrence 4-item measure: assessment of patient anxiety, worry and strong feelings associated with the return of cancer Each question in the FCR4 is rated by the patient on a scale of 1 (‘Not at all’) to 5 (‘All the time’). A cumulative score of ≥10 (60^th^ Percentile) across all 4 items is defined as ‘moderate’ fear of cancer recurrence and a cumulative score of ≥15 is defined as ‘high’ fear of cancer recurrence. | The FCR4 has been validated as an accurate measure of cancer recurrence fears in breast cancer patients that is fit for routine use in clinical services (1). |
| HADS (2) | Hospital Anxiety and Depression Scale: 14-item measure of symptoms associated with anxiety and depression. Patients report symptoms of depression and anxiety in the last week on a 4-point Likert scale, from 0 (e.g. “Not at all”) to 3 (e.g. “Most of the time”). Patients who score ≥8 in either domain are suspected to have clinical problems with anxiety and/or depression (3). | The HADS is well established and has been employed in a multitude of medical settings, including oncology, to assess patients’ mental health (4, 5). |
| EQ-5D -5L (6) | EuroQol 5 Dimension Measure of Health-related Quality of Life: The EQ-5D assesses 5 dimensions including mobility, self-care, usual activities, pain/discomfort, and anxiety/depression. The EQ-5D contains 6-items, 5 of these items assess each of the 5 dimensions and are rated on a 5-level (5L) scale. The final item is a measure of health state, which is rated on a scale of 0 (‘worst imaginable health state’) to 100 (Best imaginable health state’). | The EQ-5D is a standardised instrument of health-related quality of life, developed by the EuroQol Group (6). |
| CARE (7) | Consultation and Relational Empathy measure: 10-item person-centred process measure, assessing empathy in clinical and therapeutic consultations. Patient rate practitioners’ communication skills on a scale from 1 (‘Poor’) to 5 (‘Excellent’) in 10 communication and empathy domains. | The CARE measure has been validated for use in a secondary care setting (8). CARE has previously been employed in multiple research studies to assess the use of health practitioner empathy in cancer care (9). |
| MISS (10) | Medical Interview Satisfaction Scale: The MISS is a 29-item measure designed to assess patient satisfaction primary care consultation. Each item in the MISS is a statement relating to practitioners’ communication skills and patients are asked to rate each statement on a 5-point Likert scale (1 = Strongly disagree; 5 = Strongly agree). Only three items from the MISS will be used in this research and were selected for their specific focus on patient worry and fear. | The MISS has been validated for use in the UK primary and secondary care setting (10, 11). |

References:

1. Humphris G, Watson E, Sharpe M, Ozakinci G. Unidimensional scales for fears of cancer recurrence and their psychometric properties: the FCR4 and FCR7. Health Qual Life Outcomes. 2018;16(1):30.

2. Zigmond AS, Snaith RP. The hospital anxiety and depression scale. Acta psychiatrica Scandinavica. 1983;67(6):361-70.

3. Bjelland I, Dahl AA, Haug TT, Neckelmann D. The validity of the Hospital Anxiety and Depression Scale: An updated literature review. Journal of Psychosomatic Research. 2002;52(2):69-77.

4. Vodermaier A, Millman RD. Accuracy of the Hospital Anxiety and Depression Scale as a screening tool in cancer patients: a systematic review and meta-analysis. Support Care Cancer. 2011;19(12):1899-908.

5. Hartung TJ, Friedrich M, Johansen C, Wittchen HU, Faller H, Koch U, et al. The Hospital Anxiety and Depression Scale (HADS) and the 9-item Patient Health Questionnaire (PHQ-9) as screening instruments for depression in patients with cancer. Cancer. 2017;123(21):4236-43.

6. Herdman M, Gudex C, Lloyd A, Janssen M, Kind P, Parkin D, et al. Development and preliminary testing of the new five-level version of EQ-5D (EQ-5D-5L). Qual Life Res. 2011;20(10):1727-36.

7. Mercer SW, Maxwell M, Heaney D, Watt GCM. The consultation and relational empathy (CARE) measure: development and preliminary validation and reliability of an empathy-based consultation process measure. Family Practice. 2004;21(6):699-705.

8. Mercer SW, Murphy DJ. Validity and reliability of the CARE Measure in secondary care. Clinical Governance: An International Journal. 2008;13(4):269-83.

9. Lelorain S, Bredart A, Dolbeault S, Sultan S. A systematic review of the associations between empathy measures and patient outcomes in cancer care. Psychooncology. 2012;21(12):1255-64.

10. Meakin R, Weinman J. The 'Medical Interview Satisfaction Scale' (MISS-21) adapted for British general practice. Fam Pract. 2002;19(3):257-63.

11. Maurice-Szamburski A, Michel P, Loundou A, Auquier P, Investigators GMS. Validation of the generic medical interview satisfaction scale: the G-MISS questionnaire. Health Qual Life Outcomes. 2017;15(1):36.
