# Supplementary material for: A controlled pilot trial of a nurse-led intervention (Mini-AFTERc) to manage fear of cancer recurrence in patients affected by breast cancer
Source: Pilot Feasibility Stud. 2020 May 7;6:60. doi: 10.1186/s40814-020-00610-4 (PMC7204012; doi:10.1186/s40814-020-00610-4)
Supplement: Supplementary file 2 — Additional file 2. Sampling matrix for semi-structured interviews [file 40814_2020_610_MOESM2_ESM.docx]

Supplementary file 2: Sampling matrix for semi-structured interviews

|  |  | **Control** | **Intervention** |
| --- | --- | --- | --- |
| **Group** | Intervention | - | 100% |
|  | Control | 100% | - |
| **Age** | ≤45 yrs | 80% | 80% |
|  | <45 yrs | 20% | 20% |
| **Site** | Fife | - | 50% |
|  | Lothian | - | 50% |
|  | Highlands | 50% |  |
|  | Tayside | 50% |  |
| **FCR-4 Score** | Increase |  | 20% |
|  | Decrease |  | 60% |
|  | Stable |  | 20% |
| **Retention** | Attended | 70% | 70% |
|  | Dropped-out | 30% | 30% |
